# The Influence of BMI on Mortality and Clinical Outcomes After Burns

**DOI:** 10.3390/ebj7010012

**Published:** 2026-02-12

**Authors:** Julia Kleinhapl, Rudy Ji, Lucineia Gainski Danielski, George Golovko, Alen Palackic, Philong Nguyen, Ludwik K. Branski, Steven E. Wolf, Celeste C. Finnerty, Oscar E. Suman

**Affiliations:** 1 Department of Surgery, Division of Surgical Sciences, University of Texas Medical Branch, Galveston, TX 77555, USA; lugainsk@utmb.edu (L.G.D.); lubransk@utmb.edu (L.K.B.); oesuman@utmb.edu (O.E.S.); 2 Division of Plastic, Aesthetic and Reconstructive Surgery, Department of Surgery, Medical University of Graz, 8036 Graz, Austria; 3 John Sealy School of Medicine, Galveston, TX 77555, USA; ruji@utmb.edu (R.J.);; 4 Department of Pharmacology and Toxicology, University of Texas Medical Branch, Galveston, TX 77555, USA; gegolovk@utmb.edu; 5 Department of Hand, Plastic and Reconstructive Surgery, Burn Center, BG Trauma Center Ludwigshafen, University of Heidelberg, 67071 Ludwigshafen, Germany; alen.palackic@gmail.com; 6 Shriners Children’s Texas, Galveston, TX 77550, USA

**Keywords:** body mass index, burn injury, real-world data, clinical outcomes, obesity paradox

## Abstract

Background: Weight extremes are linked to morbidity, yet their impact on burn outcomes remains underinvestigated. Prior studies suggest an ‘obesity paradox’, showing survival benefits and better functional outcomes in obese patients. Methods: This study used the global real-world database TriNetX to assess the association between body mass index (BMI) and clinical outcomes in adult burn patients, categorized using WHO definitions. After 1:1 propensity score matching for demographics, burn severity, and smoke inhalation injury, clinical outcomes were analyzed over a six-month period following burn injury. Outcomes included mortality, sepsis, pneumonia, acute kidney injury (AKI), cardiovascular events, graft complications, skin infections, and psychological impairment. Results: After matching, 9736 patients were included in the underweight versus normal weight comparison, 72,274 in overweight versus normal weight, 71,195 in obesity versus normal weight, and 9732 in underweight versus obesity. Underweight patients were associated with higher mortality and increased risks of sepsis, pneumonia, cardiovascular events, and psychological impairment. Overweight and obese patients showed higher survival rates and overall better clinical outcome associations. Conclusions: These findings are consistent with the previously described ‘obesity paradox’ in burn care and identify underweight burn patients as a distinct high-risk subgroup.

## 1. Introduction

Overweight and obesity, defined by the World Health Organization (WHO) as a body mass index (BMI) of equal to or greater than 25 and 30 kg/m^2^, respectively, serve as the cornerstone of major healthcare issues such as cardiovascular disease, metabolic and endocrine dysfunction, and musculoskeletal long-term impairment [1,2,3,4]. As a key contributor to overall mortality and multi-morbidity beginning at an early age, obesity represents a major public health concern and remains one of the hardest-to-fight global problems, also significantly affecting children and adolescents [5,6,7,8,9].

As of August 2023, 40.3% of the adult population in the United States was categorized as overweight, and global estimates project that the number of overweight individuals will exceed 1.3 billion by the year 2030 [10,11]. On an individual level, overweight and obesity are associated with increased morbidity, higher mortality, and elevated healthcare costs that are up to 233.6% greater than those incurred by individuals of normal weight [12]. At the economic level, health industry and employers carry the burden of the overweight collectively, reflected in annual excess medical costs of $170 billion in the United States alone [13]. More specifically, Dall et al. reported that employers across common industries face more than $46 billion in direct medical expenses for employees with a BMI over 25 kg/m^2^ [14]. Ling et al. further observed an average increase of $237.55 in annual direct medical costs per overweight or obese child in the U.S., with hospitalization and prescription medication costs notably higher than those in other countries [15].

In contrast, being underweight—defined as a BMI of <18.5 kg/m^2^—increases health risks to at least the same degree as being overweight, if not more [1]. While an estimate of only 1.6% of the population aged 20 and above is classified as underweight in the United States as of 2018, higher prevalences are found in South Asia and the African continent, indicating a clear regional aspect in weight class distributions and a major health concern in developmental countries [16,17]. The prevalence of 1.6% is a result of the National Health and Nutrition Examination Surveys (NHANES), conducted by the team around Fryar et al. from the National Center for Health Statistics [16]. Their results date back to 1988 and show an overall trend of decreased underweight prevalence over time across all age groups in the U.S. population. In contrast, a worldwide trend analysis published in The Lancet in 2017 reports a peak prevalence of moderate and severe underweight in pediatric populations, particularly in India (22.7% for girls, 30.7% for boys) [17].

Complications of misalignment between nutritional demands and nutritional intake include myelosuppression, impairment of the hypothalamic–pituitary–gonadal (HPG) axis and a decreased synthesis of adenosine triphosphate (ATP) in the mitochondria. Hence, the pathophysiology of underweight translates into anemia, immune system dysfunction, impairment of the reproductive system, an overall decrease in life expectancy, and increased mortality [18,19,20,21,22,23].

The common ground of weight excesses, such as critical under- and/or overweight, hence lies in the drastic impairment of organ functions necessary to maintain physical and mental health [24,25]. While individuals with normal weight may withstand a critically ill state better, burn patients with comorbid weight excesses are inherently limited in their physiological capacity to cope with the extreme injury-induced disturbance of homeostasis across all organ systems [26]. A thermal injury places the body under tremendous metabolic demands where catabolic processes surpass anabolic counteraction [27]. While underweight patients may lack the physical capacity to meet these demands, overweight and obese patients—despite having greater physical reserves—are associated with higher risks of general complications, such as infections and anesthesia-related difficulties [28,29].

The obesity paradox has been reported to place overweight patients in a superior position, with both survival and physical function outcomes outperforming those of underweight patients in the critically ill setting and during long-term recovery after burn [29,30,31]. To explore whether this phenomenon is reflected in real-world data, we investigated this association using a global database.

## 2. Materials and Methods

### 2.1. Study Design

We conducted a retrospective cohort study using the TriNetX Analytics Network, a global federated health research platform that aggregates de-identified patient information from electronic health records. For our analysis, we accessed the TriNetX Research Network, which includes data from 110 participating healthcare organizations (HCOs) worldwide, and encompasses over 154 million patients.

Adult patients aged 18 years and older with a diagnosis of burn injury were identified using ICD-10-CM codes T20–25 and T30–T32. The first recorded burn diagnosis was defined as the index event. To assure accurate baseline characterization, data were required to include a documented BMI recorded from three months to one day prior to the index event. BMI measurements from the day of burn were excluded to avoid resuscitation-associated skewing of BMI values.

Patients were further stratified into four BMI groups, based on the World Health Organization (WHO) classification: underweight (BMI < 18.4 kg/m^2^), normal weight (BMI 18.5–24.9 kg/m^2^), overweight (BMI 25.0–29.9 kg/m^2^) and obesity (BMI ≥ 30 kg/m^2^). Four pairwise comparisons were performed independently: underweight versus normal weight (1), overweight versus normal weight (2), obesity versus normal weight (3), and underweight versus obesity (4).

**Figure 1:**
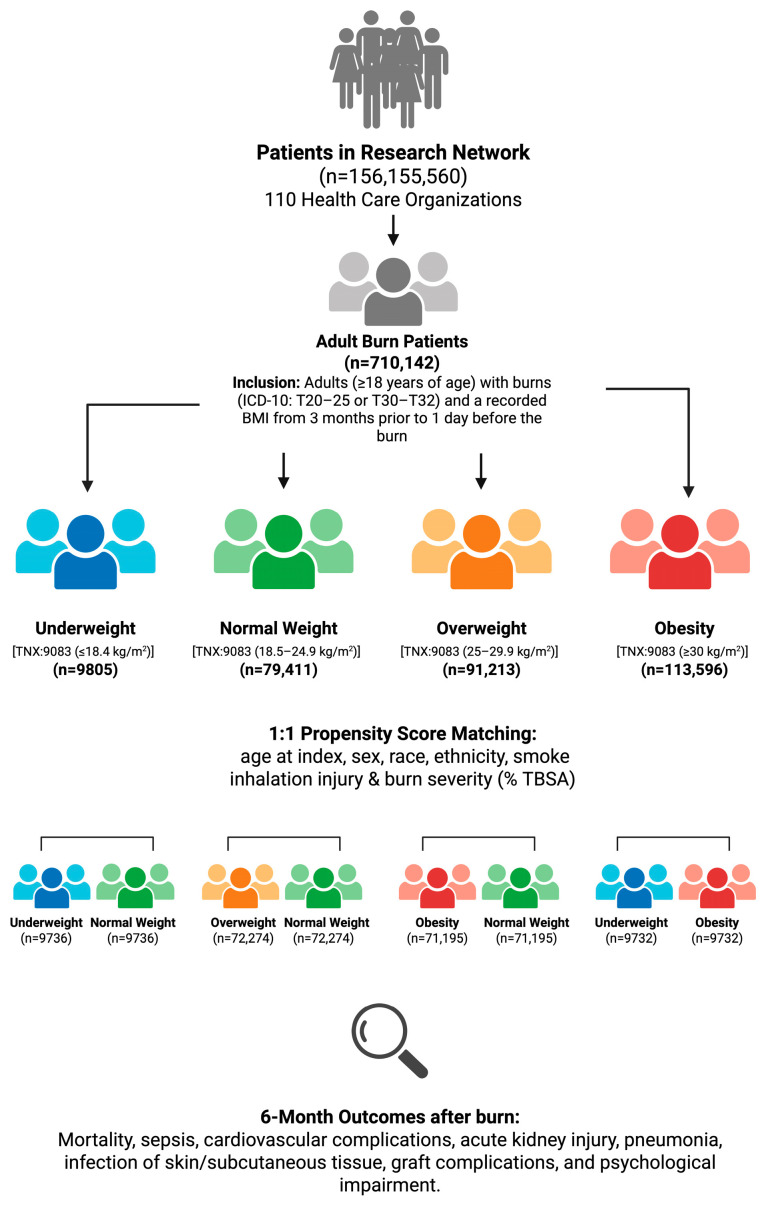
Study design and patient selection flowchart. BMI, Body Mass Index; TNX Curated, curated by the TriNetX team to harmonize related codes; TBSA, total body surface area burned. Created in BioRender. Kleinhapl, J. (2026). https://BioRender.com/f3l94z9 (accessed on 11 February 2026).

All data used in this study were de-identified, and no identifiable patient information was accessed. Hence, the study qualified as non-human subject research and did not require Institutional Review Board (IRB) approval. The study methodology is outlined in a flowchart (Figure 1).

### 2.2. Propensity Score Matching

To reduce confounding and ensure baseline similarity across the groups, 1:1 propensity score matching was performed prior to outcome analysis for each group comparison, based on the following variables: age at burn, sex, race, ethnicity, burn severity, defined as total body surface area (TBSA) burned, and presence of smoke inhalation injury (ICD-10 code J70.5). In TriNetX, propensity score matching is carried out by first computing a propensity score for every patient in the two defined cohorts. The score represents the modeled probability of belonging to the exposed cohort given the selected baseline covariates and is estimated with logistic regression in the platform backend. The resulting scores, bounded between 0 and 1, are then used to form balanced cohorts through 1 to 1 nearest neighbor greedy matching without replacement, so each exposed patient is paired to the closest control by propensity score only once. Prior to pairing, TriNetX randomly shuffles cohort order to mitigate order dependence, and applies a caliper to exclude matches that are too far apart, using a default caliper width of 0.10 pooled standard deviations in the LIVE interface.

### 2.3. Outcome Analysis

Outcomes were assessed within a six-month (180-day) period after the burn diagnosis. The primary outcomes included all-cause mortality, sepsis, pneumonia, acute kidney injury (AKI), graft complications, cardiovascular complications, and psychological impairment. Mortality was defined by recorded death or a diagnosis of ill-defined and unknown cause of mortality (ICD-10: R99). All codes used are listed in detail in Table 1. Analytical tools from the TriNetX database were used for the outcome analysis. Patients were excluded from the outcome analysis if any outcome of interest was documented prior to the burn event. Statistical results are presented as binary outcomes (risk differences with *p*-values from two-sided tests), effect sizes (risk ratios and odds ratios with 95% confidence intervals), and time-to-event outcomes reported as hazard ratios with 95% confidence intervals and log-rank *p*-values from Kaplan–Meier survival analysis. Analyses were conducted as pairwise comparisons, as simultaneous multigroup comparisons are not feasible within the TriNetX database. To account for multiple comparisons, a Bonferroni-adjusted significance threshold was applied (α = 0.05/4 = 0.0125). All statistical analyses and data extractions were performed on 14 November 2025.

## 3. Results

The TriNetX Research Network encompassed 156,155,560 patients across 110 healthcare organizations, including 710,142 adult burn patients. Initial patient counts were as follows: 9805 in the underweight cohort, 79,411 in the normal weight cohort, 91,213 in the overweight cohort, and 113,596 in the obese cohort. After propensity score matching, 9736 patients were included in each cohort for comparison 1 (underweight versus normal weight) 72,274 in comparison 2 (overweight versus normal weight), 71,195 in comparison 3 (obesity versus normal weight), and 9732 in comparison 4 (underweight versus obesity).

### 3.1. Demographics

Prior to matching, the BMI groups differed significantly in various demographic characteristics, including age at burn, burn size, presence of smoke inhalation injury, race, and ethnicity (SMD > 0.1). Matching successfully eliminated these differences and equalized group sizes in each pairwise comparison. Age and gender distribution were similar across the different groups, with an almost 50:50 sex distribution and a mean age of 47 ± 19 years in comparison 1, 46 ± 18 years in comparison 2, 46 ± 18 years in comparison 3, and 47 ± 19 in comparison 4. Most patients suffered mild burns, affecting less than 20% of the TBSA (comparison 1: 6.9% of each cohort; comparison 2: 5.2% of each cohort; comparison 3: 5.3% of obese and 4.5% of normal weight patients). While the occurrence of smoke inhalation injury differed significantly between the cohorts in comparisons 1 and 4 before matching, this significance was eliminated successfully, resulting in comparable numbers after matching (comparison 1: 2.4% vs. 2.5%; comparison 2: 1.1% of each cohort; comparison 3: 1.2% vs. 1.1%; comparison 4: 2.4% vs. 2.5%). Patient information on extent of burn, expressed as the % of TBSA burned, was not coded for all patients in the cohorts. A detailed overview of baseline characteristics across all comparisons, before and after propensity score matching, is presented in Table S1.

### 3.2. Comparison 1: Underweight vs. Normal Weight

When comparing underweight to normal weight patients, findings showed a tendency towards better outcomes in the higher weight category. Specifically, underweight patients had a significantly higher risk of mortality compared to normal weight patients (0.06% vs. 0.03%, *p* < 0.001; RR 1.99, 95% CI: 1.730,2.287; HR 2.01, 95% CI: 1.746,2.320), which was also reflected in significantly lower six-month survival (underweight: 93.26%; normal weight: 96.59%; *p* < 0.001). Sepsis was more likely to occur in underweight patients compared to normal weight burn patients (0.04% vs. 0.02%, *p* < 0.001; RR 1.69, 95% CI: 1.409, 2.015; HR 1.71, 95% CI: 1.424, 2.048). Additionally, pneumonia (0.04% vs. 0.02%, *p* < 0.001; RR 1.60, 95% CI: 1.331, 1.899; HR 1.61, 95% CI: 1.346, 1.931), acute kidney injury (0.03% vs. 0.02%, *p* = 0.002; RR 1.35, 95% CI: 1.114, 1.639; HR 1.36, 95% CI 1.120, 1.655), and cardiovascular complications (0.06% vs. 0.04%, *p* < 0.001; RR 1.52, 95% CI: 1.306, 1.771; HR 1.54, 95% CI: 1.320, 1.801) were all more commonly observed in underweight patients. We found no significant differences in the risks of graft complications or skin, psychological impairment, and subcutaneous tissue infections (Table 2).

### 3.3. Comparison 2: Overweight vs. Normal Weight

In comparing overweight patients to those categorized as normal weight, mortality was significantly higher in the latter category, reflected by an RR of 0.74 (0.02% vs. 0.03%, *p* < 0.001; RR 0.74, 95% CI: 0.695, 0.795; HR 0.73 95% CI: 0.682, 0.782). Log-rank test demonstrated a significant difference in survival (97.68% vs. 96.84%, *p* < 0.001) in the Kaplan–Meier analysis, emphasizing a clear difference in this outcome within the first six months after burn. Moreover, overweight patients showed a significantly lower risk for infectious complications such as sepsis (0.01% vs. 0.02%, *p* < 0.001; RR 0.80, 95% CI: 0.736, 0.871; HR 0.79, 95% CI: 0.724, 0.858) and pneumonia (0.02% vs. 0.02%, *p* < 0.001; RR 0.82, 95% CI: 0.757, 0.888; HR 0.81, 95% CI: 0.744, 0.874). Cardiovascular complications were less likely to occur in overweight patients compared with normal weight patients (0.03% vs. 0.03%, *p* = 0.019; RR 0.93, 95% CI: 0.869, 0.988; HR 0.91, 95% CI: 0.857, 0.975); however, this difference did not meet the Bonferroni-adjusted significance threshold. Lastly, overweight patients were at lower risk for psychological impairment, showing an 8% lower hazard over the study period compared to normal weight patients (0.07% vs. 0.07%, *p* = 0.006; RR 0.93, 95% CI: 0.889, 0.981; HR 0.92, 95% CI: 0.875, 0.968). The risks for acute kidney injury (0.02% vs. 0.02%, *p* = 0.132; RR 0.94, 95% CI: 0.868, 1.019; HR 0.93, 95% CI: 0.856, 1.006), infections of the skin and subcutaneous tissue (0.07% vs. 0.07%, *p* = 0.288; RR 1.02, 95% CI: 0.980, 1.069; HR 1.02, 95% CI: 0.972, 1.063) and graft complications (0.01% vs. 0.01%, *p* = 0.355; RR 0.95, 95% CI: 0.849, 1.060; HR 0.94, 95% CI: 0.837, 1.046) did not differ significantly between the cohorts (Table 2).

### 3.4. Comparison 3: Obesity vs. Normal Weight

Obese burn patients were more likely to experience infections of the skin or subcutaneous tissue (0.08% vs. 0.07%, *p* < 0.001; RR 1.14, 95% CI: 1.097, 1.194; HR 1.14, 95% CI: 1.087, 1.187). While they had a higher risk for skin or subcutaneous infections, obese patients had significantly lower risks for clinical outcomes than normal weight burn patients. Specifically, normal weight patients showed a 29% higher mortality risk compared to obese patients (0.02% vs. 0.03%, *p* < 0.001; RR 0.71, 95% CI: 0.660, 0.758; HR 0.69, 95% CI: 0.640, 0.737), which was also reflected in significantly worse six-month survival (97.85% in obese vs. 96.87% in normal weight patients, log-rank *p* < 0.001).

The risk of sepsis was almost 20% higher in those with normal weight (0.02% vs. 0.02%, *p* < 0.001; RR 0.83, 95% CI: 0.765, 0.905; HR 0.81, 95% CI: 0.745, 0.882) compared to obese patients. Moreover, we found a notably higher risk for complications such as pneumonia (0.02% vs. 0.02%, *p* = 0.003; RR 0.89, 95% CI: 0.821, 0.961; HR 0.87, 95% CI: 0.799, 0.937) and graft complications (0.01% vs. 0.01%, *p* = 0.002; RR 0.83, 95% CI: 0.742, 0.938; HR 0.81, 95% CI: 0.724, 0.915) in the normal weight cohort. Cardiovascular complications (0.03% vs. 0.03%, *p* = 0.50; RR 1.02, 95% CI: 0.959, 1.089) and the risk of AKI (0.02% vs. 0.02%, *p* = 0.05; RR 1.08, 95% CI: 1.000, 1.172) did not significantly differ. Finally, normal weight patients had a lower event-free survival with respect to psychological impairment (91.84% in obesity vs. 91.48% in normal weight cohort, log-rank *p* = 0.028); however, this difference did not meet the Bonferroni-adjusted significance threshold (Table 2).

### 3.5. Comparison 4: Underweight vs. Obesity

When comparing both extreme weight categories against each other, we observed survival and outcome advantages for burn patients in the obese cohort. Specifically, the mortality risk was more than twice as high in underweight patients compared to obese patients (0.06% vs. 0.02%, *p* < 0.001; RR 2.67, 95% CI: 2.282, 3.113; HR 2.77, 95% CI: 2.368, 3.247). Underweight patients had a more than 2-fold increased sepsis risk (0.04% vs. 0.02%, *p* < 0.001; RR 2.20, 95% CI: 1.814, 2.679; HR 2.30, 95% CI: 1.889, 2.804) and were more likely to experience cardiovascular complications (0.06% vs. 0.04%, *p* < 0.001; RR 1.42, 95% CI: 1.219, 1.649; HR 1.46, 95% CI: 1.254, 1.708), pneumonia (0.04% vs. 0.02%, *p* < 0.001; RR 1.99, 95% CI: 1.644, 2.412; HR 2.07, 95% CI: 1.703, 2.511), and psychological impairment (0.09% vs. 0.07%, *p* = 0.001; RR 1.25, 95% CI: 1.096, 1.421; HR 1.31, 95% CI: 1.140, 1.493). Similar to comparison 3, infections of the skin or subcutaneous tissue were more likely in obese patients (0.07% vs. 0.07%, *p* = 0.330; RR 0.94, 95% CI: 0.837, 1.062; HR 0.96, 95% CI: 0.845, 1.081); however, this difference was not significant. Graft complications (0.01% vs. 0.01%, *p* = 0.133; RR 1.25, 95% CI: 0.935, 1.657; HR 1.28, 95% CI: 0.959, 1.704) and AKI (0.03% vs. 0.02%, *p* = 0.093; RR 1.17, 95% CI: 0.973, 1.413) were more common in underweight patients as well, but without significance.

**Figure 2:**
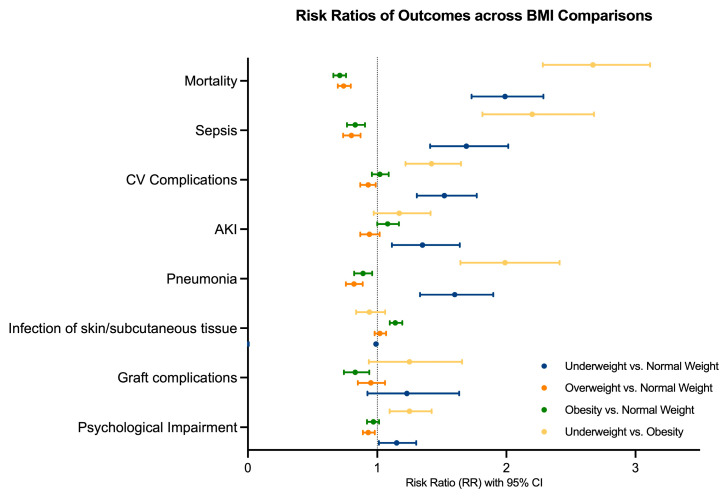
Risk ratios (RR) and 95% confidence intervals for clinical outcomes across BMI comparisons. BMI, body mass index; CV, cardiovascular; AKI, acute kidney injury. Generated using GraphPad Prism (version 10.6.1).

Overall, our results show that higher weight categories are associated with higher survival rates and lower risks of clinical complications across all comparisons, including in overweight and obese patients. Graft complications and infections of the skin or subcutaneous tissue were the least affected by excess weight, although obese patients showed a marginally increased risk for the latter, particularly when compared to normal weight patients. The strongest differences were observed in the direct comparison of the two weight extremes—underweight vs. obesity—with obese burn patients showing overall better outcomes (Figure 2).

## 4. Discussion

With both short- and long-term challenges for all organ systems, burn injuries remain one of the most debilitating threats to the body, making recovery a burdensome and prolonged trajectory [26,32,33]. For the first time, our results show that higher BMI categories were associated with higher survival and lower complication rates following burn injury across all comparisons. Hence, our study is in line with previous findings and expands existing literature on the ‘obesity paradox’ in the burn population, which describes a superiority of class I (mild) obesity in the critically ill setting after burns [31,34]. A direct comparison of elevated weight categories to both normal weight and underweight patients using large real-world data is, to our knowledge, novel and therefore contributes to the understanding of the association between BMI and burn outcomes.

The association between increased body weight and reduced short- and long-term mortality has been described in the general population, as well as among cardiovascular, oncologic, orthopedic, and emergency general surgery patients [6,35,36,37,38,39]. For example, orthopedic surgery patients demonstrated approximately half the odds of two-year mortality compared to non-obese patients [38]. In contrast, these studies report an increased risk of mortality among underweight counterparts [40]. This is in line with our findings, where the mortality risk for underweight burn patients was almost twice that of the normal weight cohort and nearly three times as high compared to obese patients. In contrast, both overweight and obese patients experienced better six-month survival compared to normal weight and underweight patients.

The United States has approximately 130 verified burn centers, but only about 22% of burned patients are treated at these facilities, according to an analysis by Zonies et al. [41]. We assume that differences in patient characteristics, treatment strategies, and facility-related resources contribute to variations in care and outcomes. An analysis on this aspect by Palmieri et al. demonstrated that a notably higher amount of severely burned patients (≥80% TBSA) are admitted to verified burn centers [42]. Most BMI-related studies originate from these specialized centers, potentially underrepresenting diverse patient populations and treatment approaches at nonverified or peripheral facilities [34,43,44]. Especially regarding BMI, regional differences exist and make comparison between single centers difficult [45,46]. Our study therefore contributes to a broader understanding of the impact of BMI on clinical outcomes and survival, which is essential for optimizing care and tailoring treatment strategies across diverse healthcare settings and BMI categories.

Existing research on this topic primarily focuses on the challenges of treating burn patients with above-average body weight. For example, Tapking et al. highlighted obesity-associated problems in burn care, emphasizing challenges in drug dosing, surgical management, and mechanical ventilation, such as determining the optimal use of positive end-expiratory pressure (PEEP) [28]. In our study, underweight patients demonstrated significantly poorer survival and clinical outcomes, highlighting the need for increased focus on this high-risk subpopulation in burn research and clinical care.

Burn size estimation during initial assessment relies on established methods such as the Lund-Browder Chart, the Rules of Nines, and the Palmar method, which do not account for excess body weight or individual variations in body composition [47]. These estimates guide the therapeutic strategy, and misestimation of burn size in patients with extreme body weights may indirectly affect treatment, leading to over- or under resuscitation, inadequate care, or inappropriate triage [47]. These limitations may be reflected in our findings, as overweight and obese patients more frequently presented with mild burns (<20% TBSA) in cases where TBSA data was available, suggesting a potential underestimation of burn size in patients with higher body mass.

Since gold-standard formulas for calculating fluid volumes, such as the Parkland formula, incorporate TBSA, this could directly affect burn resuscitation during the first 24 h after injury [48]. Established in the 1970s, the Parkland formula remains a widely used standard, although criticism has emerged regarding its accuracy [49,50]. A recent retrospective study, using data from the German Burn Registry, evaluated deviations from the Parkland formula and reported that, while excessive fluid resuscitation is generally associated with increased in-hospital mortality, this effect appears to be attenuated in obese patients [51]. In contrast, TBSA may be overestimated in underweight patients, potentially leading to over-resuscitation. This could, in part, explain the increased mortality and higher risk of infectious complications observed in our underweight patients.

While burn patients are in general inherently more susceptible to bacterial entry due to disruption or complete loss of the skin barrier, sepsis and pneumonia are common complications in the intensive care setting, further aggravated by factors such as inhalation injury or prolonged ventilation [52,53]. Although our study demonstrates a generally low incidence of sepsis and pneumonia across all groups, both complications remain strong contributors to burn-related mortality. In fact, sepsis was found to be strongly associated with TBSA, showing a 75% increase in the odds of mortality with every 10% increase in TBSA, according to a retrospective analysis by Rech et al. [54]. Pneumonia was associated with twice the mortality rate (50% vs. 22%) compared to burn patients without pneumonia in a burn center in Egypt. The same study showed that patients with concomitant inhalation injury were twice as likely to develop pneumonia post-burn [55]. The presence of inhalation injury in our study was generally low, with fewer than 3% of patients affected in each weight class and comparison group after matching.

Phung et al. investigated the relationship between BMI and pneumonia risk in a meta-analysis, reporting an 80% higher risk of community-acquired pneumonia (*p* < 0.01) and 90% higher risk of influenza-related pneumonia (*p* < 0.01) in underweight patients compared to those of normal weight [56]. In our study, we observed a clearly higher risk of pneumonia in underweight patients, and, given the six-month follow-up period, assume that these cases predominantly reflect nosocomial pneumonias.

Several studies report higher risks of infections of skin and surgical site in overweight or obese patients [57,58,59]. This aligns with our findings, where obese patients were more likely to experience skin or subcutaneous infections compared to lower weight classes, significantly so when compared to normal weight patients.

As far as psychological complications such as anxiety or depression are concerned, we observed an increased risk in underweight burn patients compared with those of normal weight (0.09% vs. 0.08%, *p* = 0.030; RR 1.15) or those with obesity (0.09% vs. 0.07%, *p* = 0.001; RR 1.25), reflecting similarly elevated risks among underweight individuals in the general population [60,61,62].

While Tolley et al. found similar results to our study, such as an increased risk for sepsis among patients with BMI < 18.5, a remarkable fact is that they had to exclude 78,000 patients because they did not have a recorded BMI, resulting in only 32% of patients for whom BMI data were available [29]. This emphasizes a strength of our study, where our high patient count consists only of patients with available BMI data. Furthermore, our sex distribution is very balanced, which is an important consideration for studies on the influence of BMI on patient outcomes, as well as a strength, given that comparable studies very often show male dominance in the gender distribution [29,31,40]. Having more male than female patients might skew results, as the sexes differ in their distribution of adipose tissue, which potentially affects morbidity and mortality [63]. Hence, studies in which females are underrepresented may draw conclusions that are not applicable to the female population.

The common ground of body weight-related literature is the reliance on BMI as a screening tool. It is of utmost importance to broaden this knowledge and bridge the gap in understanding between different body constitution types and burn outcomes. Burn patients undergo drastic changes in body composition due to burn-induced fluid shifts, systemic inflammation and prolonged hypermetabolism [26,64,65,66].

Our study is limited by the nature of the database, which is based on diagnostic and procedural coding, with coding practices varying among different institutions. Thus, TBSA coding was not available for all included patients, although it represents an essential characteristic of a burn patient, hence limiting the dataset. For the same reason, we cannot determine with certainty whether smoke inhalation injury was reported consistently across all sites, which may have influenced outcomes such as pneumonia, sepsis, and mortality. In addition, due to limitations in data granularity, severity grading of inhalation injury was not available for patients diagnosed with this condition. Comparative analysis in TriNetX is restricted to two cohorts per analysis; therefore, more complex comparisons involving multiple cohorts (e.g., one-way ANOVA) were not feasible. While this justifies our approach of pairwise cohort comparisons with subsequent Bonferroni-adjustment, it also represents a limitation of the study, as simultaneous multigroup comparisons may allow a more comprehensive evaluation of associations across BMI categories. Although we performed propensity score matching for demographics, burn severity, and smoke inhalation status, residual confounding due to comorbidities such as malignancies and metabolic or cardiovascular diseases must be considered when interpreting the results. While overweight is strongly associated with components of the metabolic syndrome, underweight may reflect underlying chronic illness or malignancy. Therefore, our results represent associations rather than causal relationships. Another point to consider is that outcomes cannot be clearly distinguished as in-hospital or post-discharge events, which underscores the need to interpret the results with caution, as post-discharge outcomes may be influenced by factors beyond the burn event itself. Lastly, we report mean age instead of median age because raw, patient-level data are not accessible in TriNetX, which provides only mean and standard deviation for age.

## 5. Conclusions

Our study demonstrates significant differences among BMI categories (underweight, normal weight, overweight, and obesity) in a broad, global dataset of adult burn patients within a six-month period following injury. We observed consistently poorer outcome associations for underweight burn patients compared with those of normal weight or those with obesity. Specifically, underweight patients were associated with increased risks of mortality and clinical complications such as sepsis, pneumonia, cardiovascular complications, acute kidney injury, and psychological impairment. Across all comparisons, higher BMI categories were associated with higher six-month survival rates and lower risks of clinical complications.

These findings underline the need for greater focus on underweight patients in burn care, as their high-risk profile may require adapted or intensified treatment strategies. Our study is the first large database analysis to compare multiple weight categories against each other in a burn population, thereby contributing to the existing literature on the obesity paradox.

Although BMI is an imperfect surrogate of body composition, our findings suggest that it may have clinical utility beyond a simple risk marker in burn care. When interpreted alongside burn severity, age, and early metabolic indicators, BMI may contribute to early risk stratification by identifying patients with limited metabolic reserve who are particularly vulnerable to the hypercatabolic response following burn injury. Importantly, the prognostic value of BMI is likely greatest when integrated into multidimensional models rather than used as a standalone predictor. Therefore, future research should incorporate causal analyses and prospective observations, as well as direct measurement of body composition and constitutional factors to further refine risk stratification and clinical management.

## Figures and Tables

**Table 1:** Diagnosis codes used for the outcome analyses. STEMI, ST-elevation myocardial infarction; NSTEMI, non-ST-elevation myocardial infarction.

Diagnosis	ICD-10 Code
**Sepsis**	
- Sepsis due to unspecified organisms	A41.9
- streptococcal sepsis	A40
- other specified sepsis	A41
- severe sepsis	R65.2
- postprocedural sepsis	T81.44
**Pneumonia**	
- pneumonia due to unspecified organisms	J18
- viral pneumonia	J12
- *Streptococcus pneumoniae*	J13
- Haemophilus influenzae	J14
- bacterial pneumonia not elsewhere classified	J15
- pneumonia due to other infectious organisms	J16
- pneumonia associated with other diseases	J17
**Acute Kidney Failure (AKI)**	N17
**Complications of skin grafts, including both allografts and autografts**	T86.82
**Cardiovascular complications**	
- heart failure	I50
- pulmonary embolism	I26
- other venous embolism and thrombosis	I82
- acute myocardial infarction	I21
- subsequent STEMI and n-STEMI	I22
- complications within 28 days of infarction	I23
- acute ischemic heart diseases	I24
- hypovolemic shock	R57.1
- paroxysmal tachycardia	I47
- atrial fibrillation and flutter	I48
- other cardiac arrhythmias	I49
**Psychological impairment**	
- mood (affective) disorders	F30–F39
- anxiety, dissociative, stress-related, somatoform, and other nonpsychotic mental disorders	F40–F48

Note: Bolded diagnoses indicate outcome categories; non-bold entries represent individual codes included within each category.

**Table 2:** Outcome parameters across different group comparisons: Underweight vs. normal weight, overweight vs. normal weight, obesity vs. normal weight, and underweight vs. obesity. UW, underweight (BMI < 18.5 kg/m^2^); NM, normal weight (BMI 18.5–24.9 kg/m^2^); OW, overweight (BMI 25–29.9 kg/m^2^); OB, obesity (BMI ≥ 30 kg/m^2^); CV, Cardiovascular; AKI, Acute kidney failure. Significance indicated by bold *p*-values (*p* < 0.0125). * Patients with the outcome before the index event (burn) were excluded from the analysis.

Outcome	Cohort	Patients in Cohort * (n)	Patients with Outcome (n)	Absolute Risk (%)	Risk Ratio (95% CI)	Odds Ratio (95% CI)	Risk Difference (%) (95% CI)	*p*-Value (Risk)	Survival Probability at End of Window (%)	*p*-Value (Log-Rank Test)	Hazard Ratio (95% CI)
**Underweight vs. Normal Weight**											
**Mortality**	UW NW	9736 9736	567 285	0.06 0.03	1.99 (1.730,2.287)	2.05 (1.774,2.371)	0.03 (0.023,0.035)	**<0.001**	93.26 96.59	**<0.001**	2.01 (1.746,2.320)
**Sepsis**	UW NW	8303 8937	299 191	0.04 0.02	1.69 (1.409,2.015)	1.71 (1.423,2.056)	0.015 (0.010,0.020)	**<0.001**	95.80 97.56	**0.000**	1.71 (1.424,2.048)
**CV Complication**	UW NW	6753 7580	374 276	0.06 0.04	1.52 (1.306,1.771)	1.55 (1.323,1.819)	0.019 (0.012,0.026)	**<0.001**	93.60 95.85	**<0.001**	1.54 (1.320,1.801)
**AKI**	UW NW	8150 8694	228 180	0.03 0.02	1.35 (1.114,1.639)	1.36 (1.117,1.659)	0.007 (0.003,0.012)	**0.002**	96.76 97.63	**0.002**	1.36 (1.120,1.655)
**Pneumonia**	UW NW	7932 8551	292 198	0.04 0.02	1.60 (1.331,1.899)	1.61 (1.343,1.936)	0.014 (0.008,0.019)	**<0.001**	95.74 97.30	**0.000**	1.61 (1.346,1.931)
**Infection of skin/subcutaneous tissue**	UW NW	7284 7728	490 524	0.07 0.07	0.99 (−0.009,0.007)	0.99 (0.873,1.126)	−0.001 (−0.009,0.007)	0.896	92.32 92.42	0.947	0.995 (0.880,1.126)
**Graft complication**	UW NW	9606 9647	104 85	0.01 0.01	1.23 (0.924,1.634)	1.23 (0.923,1.642)	0.002 (−0.001,0.005)	0.156	98.75 98.99	0.152	1.23 (0.925,1.641)
**Psychological impairment**	UW NW	5236 5934	452 336	0.09 0.08	1.15 (1.013,1.302)	1.16 (1.014,1.333)	0.011 (0.001,0.021)	0.030	89.52 91.00	0.017	1.17 (1.029,1.337)
**Overweight vs. Normal Weight**											
**Mortality**	OW NW	72,274 72,274	1430 1924	0.02 0.03	0.74 (0.695,0.795)	0.74 (0.689,0.791)	−0.007 (−0.008,−0.005)	**<0.001**	97.68 96.84	**<0.001**	0.73 (0.682,0.782)
**Sepsis**	OW NW	67,939 67,180	966 1193	0.01 0.02	0.80 (0.736,0.871)	0.80 (0.732,0.869)	−0.004 (−0.005,−0.002)	**<0.001**	98.37 97.94	**<0.001**	0.79 (0.724,0.858)
**CV Complication**	OW NW	57,513 57,829	1763 1913	0.03 0.03	0.93 (0.869,0.988)	0.92 (0.866,0.987)	−0.002 (−0.004,−0.000)	0.019	96.44 96.15	**0.006**	0.91 (0.857,0.975)
**AKI**	OW NW	65,794 65,545	1148 1216	0.02 0.02	0.94 (0.868,1.019)	0.94 (0.866,1.019)	−0.001 (−0.003,0.000)	0.132	98.00 97.86	0.069	0.93 (0.856,1.006)
**Pneumonia**	OW NW	65,353 64,868	1080 1308	0.02 0.02	0.82 (0.757,0.888)	0.82 (0.753,0.886)	−0.004 (−0.005,−0.002)	**<0.001**	98.09 97.63	**<0.001**	0.81 (0.744,0.874)
**Infection of skin/subcutaneous tissue**	OW NW	56,837 57,416	3874 3823	0.07 0.07	1.02 (0.980,1.069)	1.03 (0.979,1.074)	0.002 (−0.001,0.004)	0.288	92.46 92.56	0.474	1.02 (0.972,1.063)
**Graft complication**	OW NW	71,826 71,783	602 634	0.01 0.01	0.95 (0.849,1.060)	0.95 (0.848,1.061)	−0.000 (−0.001,0.001)	0.355	99.04 98.98	0.245	0.94 (0.837,1.046)
**Psychological impairment**	OW NW	44,249 44,635	2897 3129	0.07 0.07	0.93 (0.889,0.981)	0.93 (0.882,0.979)	−0.005 (−0.008,−0.001)	**0.006**	92.22 91.61	**0.001**	0.92 (0.875,0.968)
**Obesity vs. Normal Weight**											
**Mortality**	OB NW	71,195 71,195	1334 1886	0.02 0.03	0.71 (0.660,0.758)	0.70 (0.654,0.753)	−0.008 (−0.009,−0.006)	**<0.001**	97.86 96.87	**<0.001**	0.69 (0.640,0.737)
**Sepsis**	OB NW	66,575 66,189	984 1176	0.02 0.02	0.83 (0.765,0.905)	0.83 (0.761,0.903)	−0.003 (−0.004,−0.002)	**<0.001**	98.33 97.94	**<0.001**	0.81 (0.745,0.882)
**CV Complication**	OB NW	54,240 56,951	1829 1879	0.03 0.03	1.02 (0.959,1.089)	1.023 (0.958,1.092)	0.001 (−0.001,0.003)	0.500	96.12 96.17	0.977	1.00 (0.938,1.067)
**AKI**	OB NW	63,846 64,621	1289 1207	0.02 0.02	1.08 (1.000,1.168)	1.08 (1.000,1.172)	0.002 (0.000,0.003)	0.050	97.72 97.86	0.158	1.06 (0.978,1.144)
**Pneumonia**	OB NW	63,273 63,935	1135 1291	0.02 0.02	0.89 (0.821,0.961)	0.89 (0.818,0.961)	−0.002 (−0.004,−0.001)	**0.003**	97.93 97.64	**<0.001**	0.87 (0.799,0.937)
**Infection of skin/subcutaneous tissue**	OB NW	53,205 56,444	4074 3777	0.08 0.07	1.14 (1.097,1.194)	1.16 (1.104,1.211)	0.010 (0.007,0.013)	**<0.001**	91.56 92.55	**<0.001**	1.14 (1.087,1.187)
**Graft complication**	OB NW	70,790 70,725	511 612	0.01 0.01	0.83 (0.742,0.938)	0.83 (0.740,0.937)	−0.001 (−0.002,−0.001)	**0.002**	99.19 99.00	**0.001**	0.81 (0.724,0.915)
**Psychological impairment**	OB NW	41,091 43,347	2839 3101	0.07 0.07	0.97 (0.920,1.014)	0.96 (0.914,1.015)	−0.002 (−0.006,0.001)	0.164	91.84 91.48	0.028	0.94 (0.897,0.994)
**Underweight vs. Obesity**											
**Mortality**	UW OB	9732 9732	565 212	0.06 0.02	2.67 (2.282,3.113)	2.77 (2.357,3.249)	0.036 (0.031,0.042)	**<0.001**	93.27 97.53	**<0.001**	2.77 (2.368,3.247)
**Sepsis**	UW OB	8299 8994	299 147	0.04 0.02	2.20 (1.814,2.679)	2.25 (1.842,2.747)	0.020 (0.015,0.024)	**<0.001**	95.80 98.14	**<0.001**	2.30 (1.889,2.804)
**CV Complication**	UW OB	6749 7233	373 282	0.06 0.04	1.42 (1.219,1.649)	1.44 (1.231,1.689)	0.016 (0.009,0.023)	**<0.001**	93.61 95.59	**<0.001**	1.46 (1.254,1.708)
**AKI**	UW OB	8146 8589	228 205	0.03 0.02	1.17 (0.973,1.413)	1.18 (0.973,1.426)	0.004 (−0.001,0.009)	0.093	96.76 97.34	0.051	1.21 (0.999,1.456)
**Pneumonia**	UW OB	7929 8490	292 157	0.04 0.02	1.99 (1.644,2.412)	2.03 (1.667,2.470)	0.018 (0.013,0.023)	**<0.001**	95.74 97.90	**<0.001**	2.07 (1.703,2.511)
**Infection of skin/subcutaneous tissue**	UW OB	7281 7269	490 519	0.07 0.07	0.94 (0.837,1.062)	0.94 (0.826,1.067)	−0.004 (−0.012,0.004)	0.330	92.32 92.09	0.475	0.96 (0.845,1.081)
**Graft complication**	UW OB	9602 9652	104 84	0.01 0.01	1.25 (0.935,1.657)	1.25 (0.934,1.665)	0.002 (−0.001,0.005)	0.133	98.75 99.02	0.093	1.28 (0.959,1.704)
**Psychological impairment**	UW OB	5234 5709	452 395	0.09 0.07	1.25 (1.096,1.421)	1.27 (1.105,1.463)	0.017 (0.007,0.027)	**0.001**	89.52 91.87	**<0.001**	1.31 (1.140,1.493)

## Data Availability

The data used for this study were obtained from the TriNetX Research Network. The authors have licensed institutional access to TriNetX, and data were used under this license for the current analysis. Because TriNetX data are de-identified and subject to contractual use restrictions, they are not publicly available. Access is available to authorized users through TriNetX (https://trinetx.com, accessed on 8 February 2026).

## Supplementary Materials

The following supporting information can be downloaded at https://www.mdpi.com/article/10.3390/ebj7010012/s1, Table S1: Baseline demographics before and after propensity score matching across the different BMI group comparisons.

## Abbreviations

The following abbreviations are used in this manuscript:

BMI	Body mass index
AKI	Acute kidney injury
WHO	World Health Organization
NHANES	National Health and Nutrition Examination Survey
HPG	Hypothalamic–pituitary–gonadal
ATP	Adenosine triphosphate
HCOs	Healthcare organizations
IRB	Institutional review board
TBSA	Total body surface area
UW	Underweight
NW	Normal weight
OW	Overweight
SD	Standard deviation
SMD	Standardized mean difference
