# Supplementary material for: The Influence of BMI on Mortality and Clinical Outcomes After Burns
Source: Eur Burn J. 2026 Feb 12;7(1):12. doi: 10.3390/ebj7010012 (PMC12922039; doi:10.3390/ebj7010012)
Supplement: Supplementary file 1 [file ebj-07-00012-s001.zip › ebj-4068374-supplementary.pdf]

***Table S1*: Baseline demographics before and after propensity score matching across the different BMI group comparisons.** UW, underweight (BMI <18.5 kg/m2); NM, normal weight (BMI 18.5-24.9 kg/m2); OW, overweight (BMI 25-29.9 kg/m2); OB, obesity (BMI ≥30 kg/m2); TBSA, total body surface area; SD, standard deviation; SDM, standardized mean difference. Covariates were considered well balanced when SDM <0.1.

**UNDERWEIGHT VS NORMAL WEIGHT**	**Matching**	**Before Propensity Score Matching**				**After Propensity Score**			
**Cohort**		**Mean ± SD**	**Patients**	**% of Cohort**	**SMD**	**Mean ± SD**	**Patients**	**% of Cohort**	**SMD**
**UW**	Age at Index	46.9 ± 19.3	9,776	100%	**0.123**	46.9 ± 19.3	9,736	100%	0.001
**NW**		44.6 ± 18.6	79,140	100%		47.0 ± 19.2	9,736	100%	
**UW**	Male		4,740	48.7%	0.052		4,739	48.7%	0.004
**NW**			40,363	51.3%			4,757	48.9%	
**UW**	Female		4,992	51.3%	0.052		4,992	51.3%	0.003
**NW**			38,326	48.7%			4,976	51.1%	
**UW**	White		5,990	61.5%	0.054		5,990	61.5%	0.005
**NW**			50,460	64.1%			6,014	61.8%	
**UW**	Black or African American		1,994	20.5%	0.094		1,994	20.5%	0.002
**NW**			13,253	16.8%			2,000	20.5%	
**UW**	Hispanic or Latino		784	8.1%	0.008		784	8.1%	0.002
**NW**			6,175	7.8%			788	8.1%	
**UW**	Asian		558	5.7%	0.010		558	5.7%	0.003
**NW**			4,692	6.0%			552	5.7%	
**UW**	Not Hispanic or Latino		7,945	81.6%	0.049		7,944	81.6%	<0.001
**NW**			62,710	79.7%			7,945	81.6%	
**UW**	Burns: 10-19% TBSA (T31.1)		668	6.9%	0.076		668	6.9%	0.002
**NW**			3,992	5.1%			672	6.9%	
**UW**	Burns: 20-29% TBSA (T31.2)		274	2.8%	0.057		274	2.8%	0.004
**NW**			1,527	1.9%			268	2.8%	
**UW**	Burns: 30-39% TBSA (T31.3)		182	1.9%	0.075		182	1.9%	0.004
**NW**			769	1.0%			187	1.9%	
**UW**	Burns: 40-49% TBSA (T31.4)		134	1.4%	0.073		133	1.4%	0.007
**NW**			510	0.6%			125	1.3%	
**UW**	Burns: 50-59% TBSA (T31.5)		85	0.9%	0.060		85	0.9%	0.008
**NW**			314	0.4%			92	0.9%	
**UW**	Burns: 60-69% TBSA (T31.6)		58	0.6%	0.050		57	0.6%	0.008
**NW**			213	0.3%			51	0.5%	
**UW**	Burns: 70-79% TBSA (T31.7)		40	0.4%	0.044		39	0.4%	0.002
**NW**			135	0.2%			38	0.4%	
**UW**	Burns: 80-89% TBSA (T31.8)		18	0.2%	0.022		18	0.2%	0.005
**NW**			80	0.1%			16	0.2%	
**UW**	Burns: 90% TBSA (T31.9)		17	0.2%	0.021		17	0.2%	0.013
**NW**			76	0.1%			12	0.1%	
**UW**	Smoke inhalation injury (J70.5)		234	2.4%	**0.100**		233	2.4%	0.004
**NW**			862	1.1%			239	2.5%	
**OVERWEIGHT VS NORMAL WEIGHT**	**Matching**	**Before Propensity Score Matching**				**After Propensity Score**			
**Cohort**		**Mean ± SD**	**Patients**	**% of Cohort**	**SMD**	**Mean ± SD**	**Patients**	**% of Cohort**	**SMD**

**OW**	Age at Index	48.4 ± 17.4	90,492	100%	**0.210**	46.5 ± 17.8	72,274	100%	0.025
**NW**		44.6 ± 18.6	78,709	100%		46.1 ± 18.4	72,274	100%	
**OW**	Male		51,233	56.6%	**0.107**		37,430	51.8%	0.039
**NW**			40,363	51.3%			38,840	53.7%	
**OW**	Female		39,235	43.4%	**0.107**		34,830	48.2%	0.039
**NW**			38,326	48.7%			33,414	46.2%	
**OW**	White		58,875	65.1%	0.020		46,622	64.5%	0.006
**NW**			50,460	64.1%			46,814	64.8%	
**OW**	Black or African American		15,581	17.2%	0.010		12,947	17.9%	0.015
**NW**			13,253	16.8%			12,527	17.3%	
**OW**	Hispanic or Latino		8,913	9.8%	0.071		5,995	8.3%	0.007
**NW**			6,175	7.8%			6,129	8.5%	
**OW**	Asian		3,281	3.6%	**0.109**		3,249	4.5%	0.005
**NW**			4,692	6.0%			3,173	4.4%	
**OW**	Not Hispanic or Latino		69,761	77.1%	0.063		57,115	79.0%	0.009
**NW**			62,710	79.7%			56,842	78.6%	
**OW**	Burns: 10-19% TBSA (T31.1)		4,735	5.2%	0.007		3,740	5.2%	0.001
**NW**			3,992	5.1%			3,723	5.2%	
**OW**	Burns: 20-29% TBSA (T31.2)		1,834	2.0%	0.006		1,433	2.0%	<0.001
**NW**			1,527	1.9%			1,438	2.0%	
**OW**	Burns: 30-39% TBSA (T31.3)		874	1.0%	0.001		685	0.9%	0.004
**NW**			769	1.0%			711	1.0%	
**OW**	Burns: 40-49% TBSA (T31.4)		561	0.6%	0.004		457	0.6%	0.003
**NW**			510	0.6%			475	0.7%	
**OW**	Burns: 50-59% TBSA (T31.5)		371	0.4%	0.002		303	0.4%	0.002
**NW**			314	0.4%			296	0.4%	
**OW**	Burns: 60-69% TBSA (T31.6)		243	0.3%	<0.001		186	0.3%	0.004
**NW**			213	0.3%			202	0.3%	
**OW**	Burns: 70-79% TBSA (T31.7)		167	0.2%	0.003		124	0.2%	<0.001
**NW**			135	0.2%			125	0.2%	
**OW**	Burns: 80-89% TBSA (T31.8)		113	0.1%	0.007		77	0.1%	<0.001
**NW**			80	0.1%			76	0.1%	
**OW**	Burns: 90% TBSA (T31.9)		88	0.1%	<0.001		69	0.1%	0.002
**NW**			76	0.1%			73	0.1%	
**OW**	Smoke inhalation injury (J70.5)		984	1.1%	0.001		797	1.1%	0.003
**NW**			862	1.1%			820	1.1%	
**OBESITY VS NORMAL WEIGHT**	**Matching**		**Before Propensity Score Matching**				**After Propensity Score**		
**Cohort**		**Mean ± SD**	**Patients**	**% of Cohort**	**SMD**	**Mean ± SD**	**Patients**	**% of Cohort**	**SMD**
**OB**	Age at Index	48.7 ± 16.2	112,746	100%	**0.236**	46.3 ± 17.5	71,195	100%	0.029
**NW**		44.6 ± 18.6	78,709	100%		45.8 ± 18.4	71,195	100%	
**OB**	Male		52,159	46.3%	**0.101**		35,968	50.5%	0.017
**NW**			40,363	51.3%			35,351	49.7%	
**OB**	Female		60,567	53.7%	**0.101**		35,216	49.5%	0.017
**NW**			38,326	48.7%			35,826	50.3%	
**OB**	White		73,603	65.3%	0.025		47,414	66.6%	0.010

**NW**			50,460	64.1%			47,080	66.1%	
**OB**	Black or African American		23,699	21.0%	**0.107**		12,307	17.3%	0.032
**NW**			13,253	16.8%			13,167	18.5%	
**OB**	Hispanic or Latino		10,176	9.0%	0.042		6,136	8.6%	0.005
**NW**			6,175	7.8%			6,029	8.5%	
**OB**	Asian		1,596	1.4%	**0.243**		1,596	2.2%	0.001
**NW**			4,692	6.0%			1,610	2.3%	
**OB**	Not Hispanic or Latino		86,725	76.9%	0.067		55,941	78.6%	0.003
**NW**			62,710	79.7%			55,846	78.4%	
**OB**	Burns: 10-19% TBSA (T31.1)		4,303	3.8%	0.061		3,773	5.3%	0.038
**NW**			3,992	5.1%			3,196	4.5%	
**OB**	Burns: 20-29% TBSA (T31.2)		1,656	1.5%	0.036		1,400	2.0%	0.017
**NW**			1,527	1.9%			1,240	1.7%	
**OB**	Burns: 30-39% TBSA (T31.3)		871	0.8%	0.022		683	1.0%	0.004
**NW**			769	1.0%			655	0.9%	
**OB**	Burns: 40-49% TBSA (T31.4)		560	0.5%	0.020		456	0.6%	0.005
**NW**			510	0.6%			427	0.6%	
**OB**	Burns: 50-59% TBSA (T31.5)		338	0.3%	0.017		270	0.4%	0.003
**NW**			314	0.4%			257	0.4%	
**OB**	Burns: 60-69% TBSA (T31.6)		217	0.2%	0.016		178	0.3%	<0.001
**NW**			213	0.3%			177	0.2%	
**OB**	Burns: 70-79% TBSA (T31.7)		136	0.1%	0.013		106	0.1%	0.003
**NW**			135	0.2%			113	0.2%	
**OB**	Burns: 80-89% TBSA (T31.8)		112	0.1%	0.001		84	0.1%	0.007
**NW**			80	0.1%			68	0.1%	
**OB**	Burns: 90% TBSA (T31.9)		79	0.1%	0.009		55	0.1%	0.004
**NW**			76	0.1%			63	0.1%	
**OB**	Smoke inhalation injury (J70.5)		1,015	0.9%	0.020		824	1.2%	0.010
**NW**			862	1.1%			749	1.1%	
**UNDERWEIGHT VS OBESITY**	**Matching**		**Before Propensity Score Matching**				**After Propensity Score**		
**Cohort**		**Mean ± SD**	**Patients**	**% of Cohort**	**SMD**	**Mean ± SD**	**Patients**	**% of Cohort**	**SMD**
**UW**	Age at Index	46.9 ± 19.3	9,737	100%	**0.101**	47.0 ± 19.3	9,732	100%	0.004
**OB**		48.7 ± 16.2	112,746	100%		46.9 ± 19.0	9,732	100%	
**UW**	Male		4,740	48.7%	0.048		4,738	48.7%	0.009
**OB**			52,159	46.3%			4,783	49.1%	
**UW**	Female		4,992	51.3%	0.049		4,989	51.3%	0.009
**OB**			60,567	53.7%			4,945	50.8%	
**UW**	White		5,990	61.5%	0.078		5,990	61.5%	0.002
**OB**			73,603	65.3%			5,998	61.6%	
**UW**	Black or African American		1,994	20.5%	0.013		1,994	20.5%	0.005
**OB**			23,699	21.0%			1,973	20.3%	
**UW**	Hispanic or Latino		784	8.1%	0.035		784	8.1%	0.008
**OB**			10,176	9.0%			804	8.3%	
**UW**	Asian		558	5.7%	**0.234**		553	5.7%	0.002

**OB**		1,596	1.4%		558	5.7%	
**UW**	Not Hispanic or Latino	7,945	81.6%	**0.116**	7,940	81.6%	0.004
**OB**		86,725	76.9%		7,923	81.4%	
**UW**	Burns: 10-19% TBSA (T31.1)	668	6.9%	**0.136**	668	6.9%	0.014
**OB**		4,303	3.8%		704	7.2%	
**UW**	Burns: 20-29% TBSA (T31.2)	274	2.8%	0.093	274	2.8%	0.004
**OB**		1,656	1.5%		281	2.9%	
**UW**	Burns: 30-39% TBSA (T31.3)	182	1.9%	0.096	181	1.9%	0.031
**OB**		871	0.8%		142	1.5%	
**UW**	Burns: 40-49% TBSA (T31.4)	134	1.4%	0.091	133	1.4%	0.004
**OB**		560	0.5%		129	1.3%	
**UW**	Burns: 50-59% TBSA (T31.5)	85	0.9%	0.075	84	0.9%	0.017
**OB**		338	0.3%		69	0.7%	
**UW**	Burns: 60-69% TBSA (T31.6)	58	0.6%	0.064	57	0.6%	0.019
**OB**		217	0.2%		44	0.5%	
**UW**	Burns: 70-79% TBSA (T31.7)	40	0.4%	0.056	38	0.4%	0.010
**OB**		136	0.1%		32	0.3%	
**UW**	Burns: 80-89% TBSA (T31.8)	18	0.2%	0.023	17	0.2%	0.013
**OB**		112	0.1%		12	0.1%	
**UW**	Burns: 90% TBSA (T31.9)	17	0.2%	0.030	17	0.2%	<0.001
**OB**		79	0.%		17	0.2%	
**UW**	Smoke inhalation injury (J70.5)	234	2.4%	**0.118**	231	2.4%	0.007
**OB**		1,015	0.9%		241	2.5%	
